# Riboproteomics: A versatile approach for the identification of host protein interaction network in plant pathogenic noncoding RNAs

**DOI:** 10.1371/journal.pone.0186703

**Published:** 2017-10-26

**Authors:** Sonali Chaturvedi, A. L. N. Rao

**Affiliations:** Department of Microbiology & Plant Pathology, University of California, Riverside, California, United States of America; USDA Agricultural Research Service, UNITED STATES

## Abstract

Pathogenic or non-pathogenic small (17 to 30 nt) and long (>200 nt) non-coding RNAs (ncRNAs) have been implicated in the regulation of gene expression at transcriptional, post-transcriptional and epigenetic level by interacting with host proteins. However, lack of suitable experimental system precludes the identification and evaluation of the functional significance of host proteins interacting with ncRNAs. In this study, we present a first report on the application of riboproteomics to identify host proteins interacting with small, highly pathogenic, noncoding satellite RNA (sat-RNA) associated with Cucumber mosaic virus, the helper virus (HV). RNA affinity beads containing sat-RNA transcripts of (+) or (-)-sense covalently coupled to cyanogen bromide activated sepharose beads were incubated with total protein extracts from either healthy or HV-infected *Nicotiana benthamiana* leaves. RNA-protein complexes bound to the beads were eluted and subjected to MudPIT analysis. Bioinformatics programs PANTHER classification and WoLF-PSORT were used to further classify the identified host proteins in each case based on their functionality and subcellular distribution. Finally, we observed that the host protein network interacting with plus and minus-strand transcripts of sat-RNA, in the presence or absence of HV is distinct, and the global interactome of host proteins interacting with satRNA in either of the orientations is very different.

## Introduction

In a given cell or organism, biological and physiological processes are regulated by protein-protein interactions (PPI) [1]. Proteomics, the study involving the characterization of the protein content of the genome of a given biological system, offers the potential value to understand the complex nature of the cell or organism [2]. The advent of state-of-the-art proteomics approaches such as 2D electrophoresis, shotgun proteomics, MuDPIT, protein array, etc. in conjunction with bioinformatics tools can be applied to advance our understanding of how PPI occur in tissues, cells or organelles. In contrast to mRNAs capable of synthesizing proteins, despite incapacitated to translate any proteins, non-coding RNAs (ncRNA) of host origin can play an important role in gene expression at transcriptional, post-transcriptional and epigenetic level. When ncRNAs are of non-host origin, they successfully infect the eukaryotic cells. Several small ncRNAs have been shown to be highly pathogenic to plants. These include viroids and virus-associated satellite RNAs (sat-RNA) [3, 4]. A sat-RNA associated with Cucumber mosaic virus (CMV) of 336 nucleotides (nt) long is a ncRNA dependent on CMV, the helper virus (HV), for replication as well as encapsidation. sat-RNA has a 5’-terminal cap and a 3’-terminal-CCC^OH^ [3]. sat-RNA is extensively base-paired, making sat-RNA highly stable in the absence of HV up to two weeks. Since sat-RNA being an important plant pathogen [2, 5], it is imperative to understand its biology [4].

**Fig 1:**
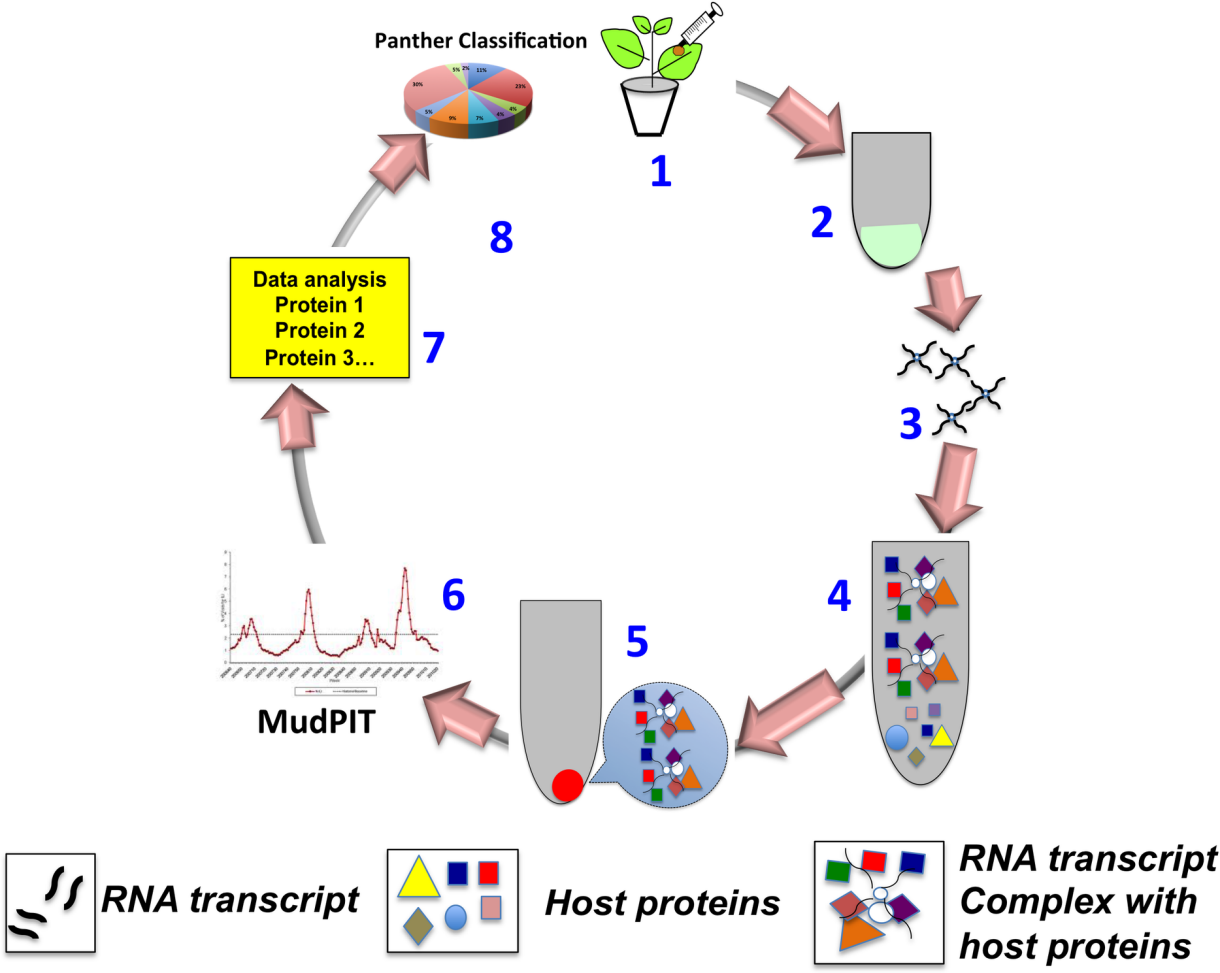
Riboproteomics. Schematic representation of various steps involved in performing the riboproteomcs. *Step1*: Infect the desired plant spp. with an RNA virus under study; healthy plant would serve as a control; *Step 2*: Total proteins are isolated from infected and healthy leaves as described under Experimental section. *Step 3*: RNA affinity beads are prepared by covalently coupling approximately 100 μg of RNA transcripts of desired polarity to cynogen bromide (CNBR)-activated sepharose beads; *Step 4*. The RNA-linked sepharose beads are incubated at 4°C for 2 hr with total proteins preparation (from Step 2) of either infected or healthy leaf tissue. *Step 5*: RNA-protein complexes bound to the beads are then eluted. *Steps 6 and 7*: The eluted protein samples are analyzed by MudPIT and identified. *Step 8*: Identified proteins are subjected to further classification (eg. Panther classification).

Since RNA viruses have evolved to possess smaller genomes encoding a limited number of genes [5] [6] [7], they exploit host proteins for sustained replication and other events to establish a successful infection [8] [3]. For example, tombusviruses have a genome size of 4.7 kb, encoding only five genes [9]. However, these viruses have been shown to hijack several host proteins to perform replication, assembly and movement and causing serious diseases in plants. Identification of host proteins interacting with virus-encoded proteins leading to a change in global protein distribution in cells infected with eukaryotic viruses involves the application of 2D-gel electrophoresis followed by mass spectrometric analysis [10]. By contrast, in the case of ncRNAs such as sat-RNA, where no protein is synthesized, studying host proteins involved in the infection lifecycle of sat-RNA can be a challenge. Recently, application of riboproteomics approach allowed the identification of viral RNA-protein interactome regulating the replication of a Norovirus [11]. In this study, we extended this riboproteomics approach (Fig 1) to pull down host proteins specifically interacting with positive or negative sense sat-RNA transcripts either in the presence or absence of the HV. Results demonstrate a drastic difference in the enrichment of host proteins in each case. This information would help to delineate host factors interacting with sat-RNA in the absence of its HV and also provide information on how the proteome of sat-RNA infected leaf changes when challenged with the HV.

## Materials and methods

### CMV strain, agroinfiltration and preparation of cell extract

Throughout this study, we used Q strain of CMV (Q-CMV) [12] and its sat-RNA (Qsat-RNA) [3]. Characteristic features of *Agrobacterium-*based T-DNA constructs of the three genomic RNAs of Q-CMV and Qsat-RNA are as previously described [12, 13]. Wild-type *Nicotiana benthamiana* leaves were infiltrated with CMV agrocultures [14] [15]. Either healthy or four days post infiltrated (dpi) *N*. *benthamiana* leaves with CMV agrotransformants were used to prepare the total protein extract. Briefly, leaves were ground in liquid nitrogen, and total protein was extracted in 3 volumes of extraction buffer (20 mM Tris-Cl [pH 7.5], 300 mM NaCl, 5 mM MgCl_2_, 5 mM DTT, 1% plant protease inhibitor [Sigma, USA]). The liquid extract was centrifuged at 12,000 rpm for 15 minutes at 4°C, and the supernatant was collected for subsequent experiments.

### Preparation of sat-RNA affinity beads

sat-RNA affinity beads are prepared as described previously [11]. Briefly, sat-RNA (+) and (-)-sense transcripts were synthesized *in-vitro using* MEGAscript T7 Transcription Kit (Invitrogen). Affinity beads containing 100 μg of either sat-RNA (+) or (-)-sense transcripts were covalently coupled to cyanogen bromide (CNBR)-activated sepharose beads. Briefly, a 125 μl preparation of pre-swollen CNBR activated Sepharose beads (Sigma) were equilibrated with 200 mM MES (pH 6.0). Then, 100 μg of (+) or (-)-sat-RNA transcripts prepared above were added to the solution containing equilibrated sepharose beads and incubated overnight at 4°C with gentle mixing. These beads were then washed three times with 100 mM Tris (pH 8.0) and continued to incubate in 100 mM Tris (pH 8.0) for 1 hr at 4°C. sat-RNA-linked Sepharose beads were washed three times in RNA binding buffer containing 50 mM HEPES (pH 7.6), 50 mM KCl, 5 mM MgO-acetate, 125 mM NaCl, 2 mM DTT and 10% glycerol.

### Enrichment of RNA binding proteins using RNA affinity beads and MudPIT analysis

Approximately 10 μg of total protein extract prepared from either healthy or CMV-infected *N*. *benthamiana* leaves was added to RNA affinity beads prepared above along with 100 μg yeast RNA, 1 mM ATP, 1mM GTP and 100 U Ribonuclease inhibitor (Sigma, U.S.A). The resulting mixture was incubated at 4°C for 3 hr with gentle mixing. RNA affinity beads were washed three times with RNA binding buffer at 4°C. Proteins bound to RNA affinity beads were eluted by treating the RNA affinity beads with RNAse A for 30 minutes, followed by centrifugation, supernatant was further subjected to trypsin digestion and MudPIT analysis. For protein identification, MASCOT MS/MS Ions search tool was used to search manually against National Center for Biotechnology Information (NCBI) non-redundant database.

### Bioinformatic tools

Panther Classification (http://www.pantherdb.org) database [16] was used for analyzing the identified proteins based on their biological functions. Gene ontology terms were determined for each protein, and statistical significance was obtained by *p* values, where *p* values < 0.05 were considered significant. Functionalities, which were seen as significant, were based on several biological functions essential for the replication of a positive sense RNA virus, like nucleic acid binding, catalytic activity, and others. For subcellular localization, WoLF PSORT (http://www.wolfpsort.seq.cbrc.jp) program [17] was used. Application of STRING (Search Tool for the *R*etrieval of *I*nteracting *G*enes) database [18] allowed the prediction of functional protein interactions in *Arabidopsis thaliana* infected with sat RNA (+) or (-) in the presence and absence of its HV. The STRING analysis was performed with confidence setting of 0.9 for the *A*. *thaliana* database.

## Results and discussion

### Distribution of host proteins interacting with (+) or (-)-sat-RNA by itself or in the presence of HV

**Fig 2:**
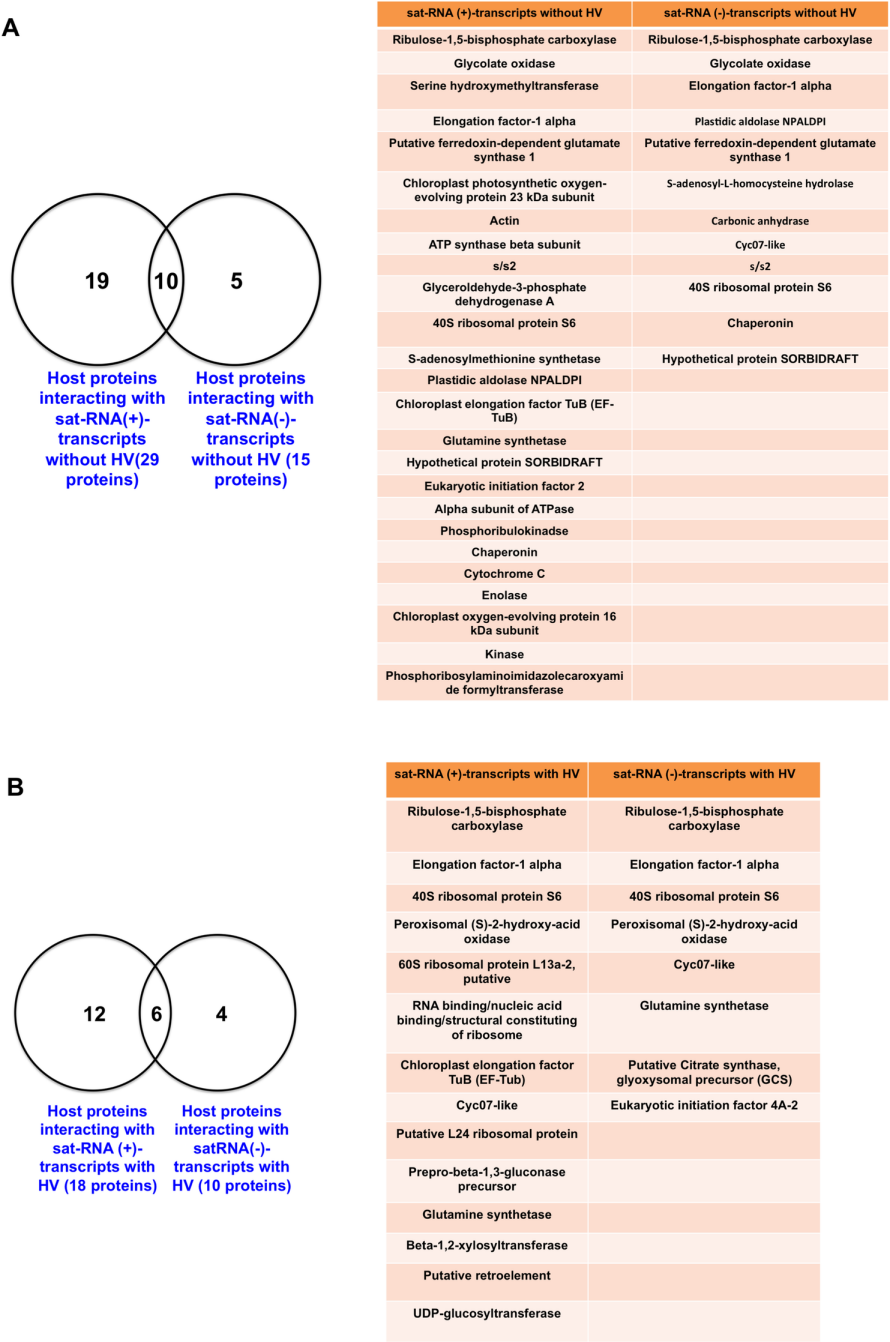
Distribution of host proteins interacting with sat-RNA affinity columns for (A) sat-RNA (+) and (B) sat-RNA (-) by itself or in the presence of HV. (A) Venn Diagram of host proteins interacting with (+)-sat-RNA transcripts, where 25 host proteins interacted with (+)-sat-RNA and 11 host proteins with (+)-sat-RNA in the absence of HV. Table shown the list of host proteins interacting with sat-RNA (+) by itself, or in the presence of HV. (B) Venn Diagram of host proteins interacting with (-)-sat-RNA transcripts, where 14 host proteins interacted with (-)-sat-RNA and 8 host proteins with (-)-sat-RNA in the absence of HV. Table shows the list of host proteins interacting with sat-RNA (-) by itself, or in the presence of HV.

A fundamental characteristic feature of (+)-stranded RNA viruses pathogenic to eukaryotic cells is that the newly synthesized (+)-strand accumulates in copious quantities as the infection progresses [19]. It is assumed that complementary viral (-)-strands that serve as templates for (+)-strands accumulate at significantly lower level than (+)-strands [20]. However, (-)-strand RNAs are the most efficient templates for (+)-strand synthesis, since each (-)-strand serves as a template for 100-fold excess of (+) strands [21]. Most importantly, a plethora of host proteins has been identified to play a cardinal role in the replication of a wide-range of RNA viruses [22]. In the case of a sat-RNA, which down-regulates the replication of HV and modulate symptom expression [23], no information is available on host proteins interacting with (+) or (-)-sat-RNA. To shed a light on the number of host proteins interacting with (+) or (-)-sat-RNA, sat-RNA affinity beads were prepared by covalently linking (+) or (-)-sat-RNA transcripts to cyanogen bromide activated sepharose beads. Further, leaf extract from healthy *N*. *benthamiana* was mixed with RNA affinity beads for (+) or (-)-sat-RNAs, followed by MudPIT analysis to identify host proteins interacting with (+) or (-)-sat-RNA. Using Riboproteomics approach (Fig 1), first we identified the number of host proteins interacting with (+) and (-)-sat-RNA in the absence of HV. Results are summarized in Venn diagrams and Tables Fig 2A and 2B. The number of host proteins interacting with (+)-sat-RNA in the absence of HV was 29 (Venn diagram shown in Fig 2A). This number was decreased to 15 for (-)-sat-RNA (Fig 2A). Of these, 10 proteins were commonly shared between (+) and (-)-sat-RNA (Fig 2A). Table in Fig 2A summarizes a selected list of host proteins identified to interact with (+) or (-)-sat-RNA. Host proteins that exclusively interacted with (-)-sat-RNA include S-adenosyl-L-homocysteine hydrolase, Carbonic anhydrase and Cyc07-like (Table in Fig 2A).

Despite the lack of recognizable sequence similarity between sat-RNA and HV genome, sat-RNA competitively hijacks HV replicase for catalyzing its replication [23], suggesting a competition for host proteins between sat-RNA and HV exists. Therefore, a list of host proteins interacting with sat-RNA in the absence of HV (Table in Fig 2A) would not provide the host proteome scenario involved in the regulation of sat-RNA replication. Consequently, to isolate and identify host proteins interacting with (+) or (-)-sat-RNA in the presence of HV, *N*. *benthamiana* leaves were infiltrated with 0.1 OD of QCMV (i.e. the HV) agrocultures. At 4 days post infiltration (dpi), leaves were ground in liquid nitrogen, and leaf extract was prepared, followed by precipitation of host proteins using (+) or (-)-sat-RNA affinity beads (Fig 1). Results are summarized in Venn diagram and Table in Fig 2(B). Interestingly, compared to the absence, presence of the HV has led to a decrease in the number of host proteins to 18 interacting with (+)-sat-RNA and to 10 for (-)-sat-RNA (compare Venn diagrams shown in Fig 2A and 2B), suggesting a shift in the proteome of (+) and (-)-sat-RNA in the presence of HV. HV-dependent replication of sat-RNA results in the accumulation ratio of sat-RNA (+):(-) 2–3:1[13, 24], implying that a distinct mechanism regulates the synthesis of (+) and (-)-strand. Therefore, the observed shift in the proteome (Fig 2A and 2B) suggests that exclusive host proteins associated with (+) or (-)-sat-RNA likely to play a significant role in maintaining the optimal ratio of (+) and (-)-strand progeny RNA.

### Functional classification, cellular distribution, interactome of proteome for (+) or (-)-sat-RNA in the absence and presence of HV

**Fig 3:**
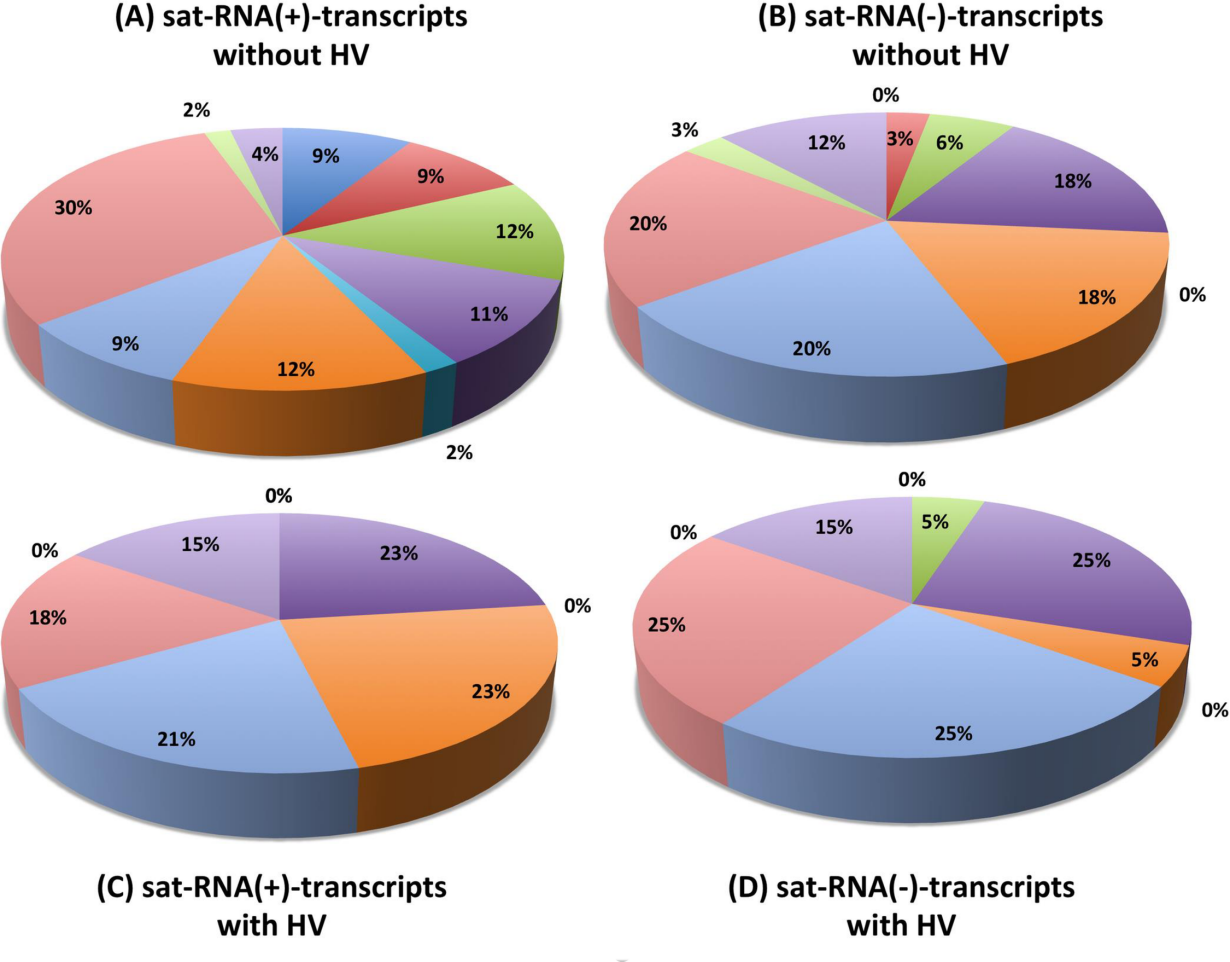
Pie-chart for functional classification of host proteins interacting with sat-RNA (+) or (-) either (A, B) in the absence or (C, D) in the presence of the HV. See Table 1 for details.

To understand further the biological relevance of the host proteins recovered by the riboproteomics approach (Fig 1), PANTHER classification system was used to classify according to the gene ontology and protein categories in which they are present. Host proteins were classified into ten biological processes groups (Fig 3, Table 1). For (+)-sat-RNA in the absence of HV, 17 proteins had a catalytic activity while only 1 of enriched proteins were involved in protein transport or chaperone activity whereas for (-)-sat-RNA 7 proteins exhibited catalytic activity and no proteins with transport activity were recovered. By contrast, in the presence of HV, the number of enriched host proteins with specific functionality has changed. For example, for (+)-sat-RNA, none of the host proteins with assigned functions in transmembrane transport activity, transporter activity, nucleobase, nucleoside, nucleotide, and nucleic acid metabolic process, protein transport or chaperone activity were recovered; whereas ~50% of the recovered proteins exhibited with assigned functions in binding, or nucleic acid binding function (Table 1, Fig 3). For (-)-sat-RNA enriched host proteins involved in binding, translation factor activity, or catalytic activity were ~50%, while no proteins having a function in transmembrane transport activity, transporter, protein transport, or chaperone activity were recovered (Table 1, Fig 3).

**Table 1:** Classification of host proteins identified by riboproteomics on the basis of their functionality.

**Sr No.**	**Function**	**(+)satRNA**	**(-) satRNA**	**(+)satRNA + CMV**	**(-) satRNA + CMV**
1	TRANSMEMBRANE TRANSPORT ACTIVITY	5, 0	0	0	0
2	TRANSPORTER	5, 0	1, 0	0	0
3	NUCLEOBASE, NUCLEOSIDE, NUCLEPTODE AND NUCLEIC ACID METABOLIC PROCESS	7, 3.07E-182	2, 0	0	1, 6.1E-177
4	BINDING	6, 4.16E-183	6, 3.5E-63	9, 1.33E-101	5, 1.18E124
5	PROTEIN TRANSPORT	1,0	0	0	0
6	NUCLEIC ACID BINDING	7, 1.71E-105	6, 3.5E-62	9, 1.33E-101	1, 1.18E-124
7	TRANSLATION FACTOR ACTIVITY	5, 2.4E-105	7, 3E-63	8, 1.5E-101	5, 1.18E-124
8	CATALYTIC ACTIVITY	17, 4.41E-155	7, 1.12E-70	7, 1.18E-137	5, 1.22E-177
9	CHAPERONE	1, 1.1e-295	1,0	0	0
10	RIBOSOMAL PROTEIN	2, 6e-105	4, 5.25e-63	6, 2.0e-101	3, 1.96e-124

Numbers in 4–7 columns represent number of host proteins and second number represents error rate belonging to the specific function.

**Fig 4:**
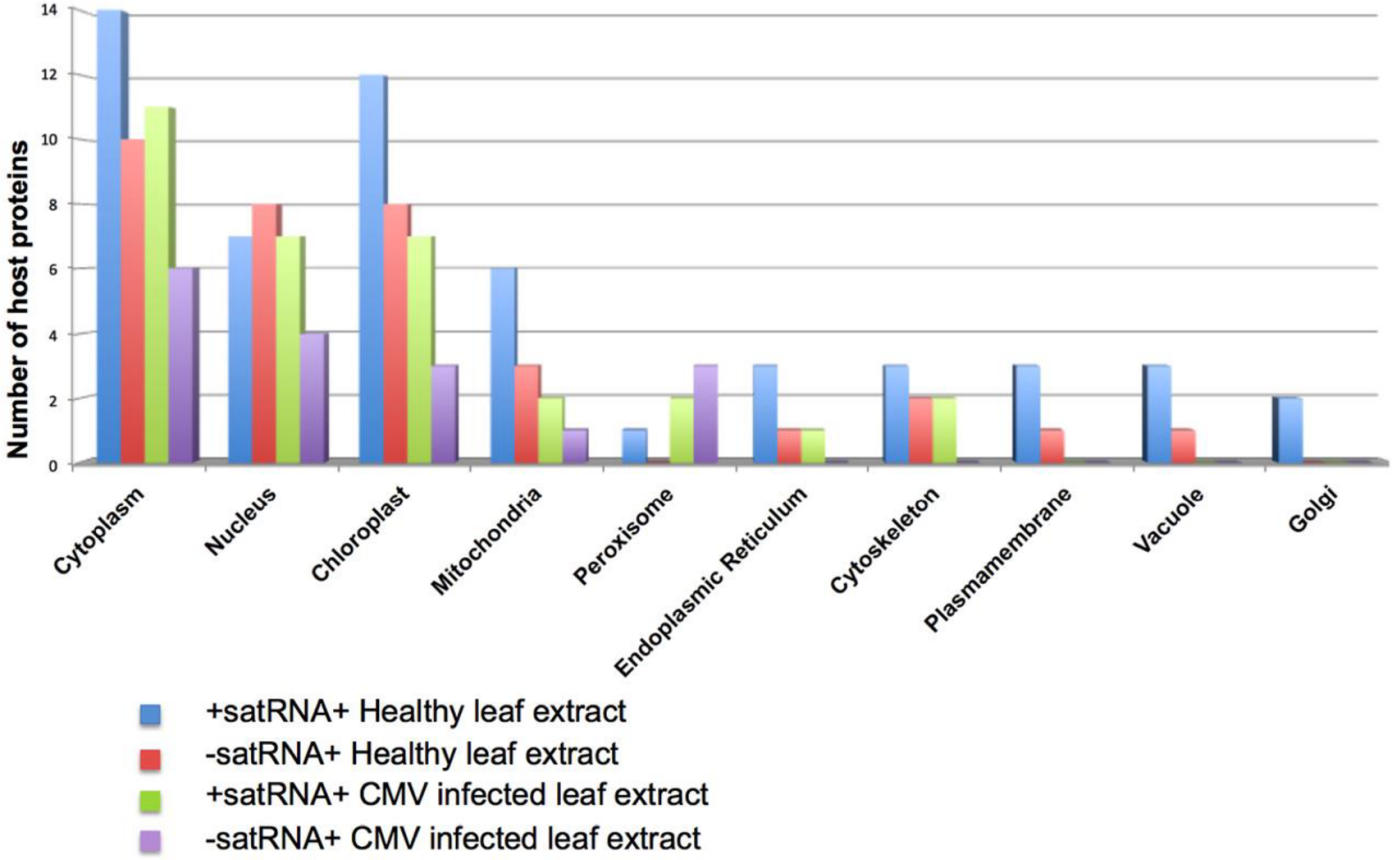
Subcellular distribution patterns of host proteins interacting with sat-RNA (+) or (-) affinity beads either in the presence or absence of CMV. WoLF- PSORT program was used to find subcellular localization of host proteins interacting with sat-RNA.

Subcellular localization of proteins plays a significant role in the replication and overall biology of the virus. Therefore, for classifying host proteins based on their subcellular localization sites, WoLF PSORT program was used. WoLF PSORT classifies proteins into more than 10 localization sites, along with dual localizations for proteins having localization signal for more than one site in the cell [17]. Results are shown in Fig 4. Classification of proteins based on their localization suggests that enriched proteins for (+)-sat-RNA either in the presence or absence of HV have predominantly distributed in the cytoplasm, nucleus and the chloroplast (Fig 4). A similar trend was observed for (-)-sat-RNA as well (Fig 4). Unlike HV whose replication is entirely cytoplasmic [25] sat-RNA has two distinct subcellular phases: HV-independent nuclear phase and HV-dependent cytoplasmic phase [13, 26]. Consequently, it is imperative to identify the number of host proteins binding to (+)-sat-RNA having nuclear localization signals. It was observed that out of total number of enriched proteins, 24% of proteins interacting with (+)-sat-RNA had a nuclear localization signal and this number was nearly doubled for (-)-sat-RNA. The percentage of host proteins interacting with sat-RNA in the presence of HV remained unaltered, which for (+)-sat-RNA was 38% and (-)-sat-RNA 40%.

**Fig 5:**
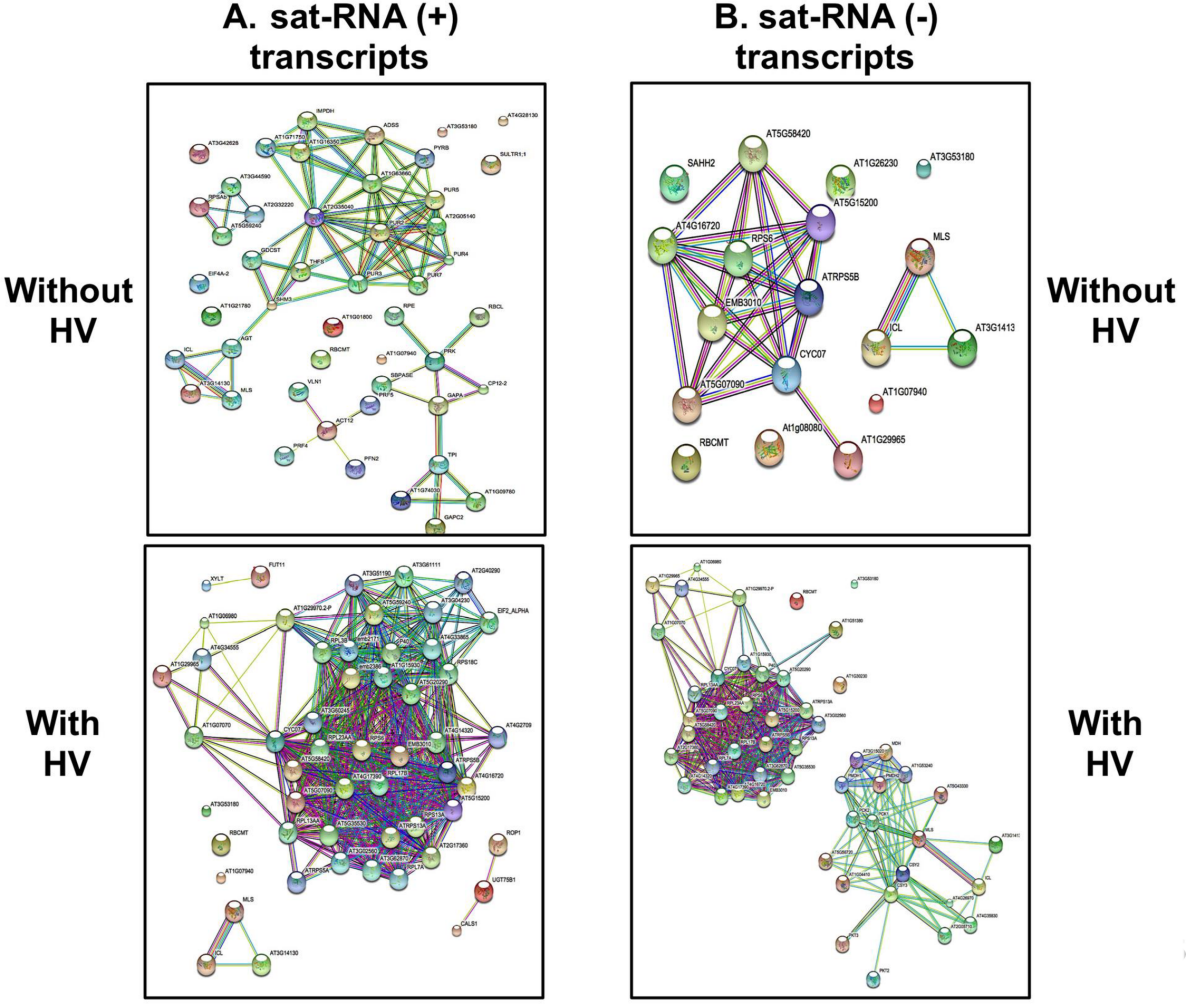
Schematic visualization of functional protein interaction network of *A*. *thaliana* using STRING when (A) sat-RNA (+) or (B) (-)-transcripts are allowed to interact with host protein network either in the absence or presence of the HV. Host protein interactome maps determined above confidence setting of 0.9 using STRING database.

Understanding system-wide cellular functions require an analysis of all functional interactions among proteins. Application of STRING database is a promising approach to evaluate the functional interaction networks of protein in a given host would change upon infection by a given pathogen [18]. Unfortunately, STRING database is available for *Arabidopsis thaliana* but not for *N*. *benthamiana*. Since *A*. *thaliana* is susceptible to CMV and its sat-RNA, using the confidence setting of 0.9 for *A*. *thaliana* database, we constructed protein interaction networks focusing protein complexes to explore novel interactions linked to sat-RNA in the presence and absence of HV. To this end, we envision a drastic shift in the functional interaction networks of *N*. *benthamiana* proteome in the presence of HV since the addition of HV has a profound influence on the variation on the proteome (Fig 2A and 2B). Results shown in Fig 5 suggest how the functional protein interaction network differs in each case. As expected, compared to the absence, in the presence of HV the protein interaction pathways are densely connected because of the availability of protein networks of both sat-RNA and HV. In addition, in Table 2, we show a selected set of closely related host proteins of *Nicotiana* sp to those of *A*. *thaliana* involved in interaction networks (Fig 5). Experiments are in progress to evaluate the functional significance of these proteins in the replication and pathogenicity of sat-RNA.

**Table 2:** Host proteins of *A*. *thaliana* and *Nicotiana spp*. involved in interaction networks.

**Sr.No**	**Gene Symbol**	**Gene Name for** ***Arabidopsis thaliana*** **Proteins**	**Gene Symbol for Closely Related *Nicotiana spp*. Proteins**	**Gene Names for** **Closely Related** ***Nicotiana spp*. Proteins**
**A. sat-RNA (+) transcripts without HV**				
1	AT3G42628	Phosphoenolpyruvate Carboxylase-related / PEP Carboxylase-like Protein	LOC107777405	Phosphoenolpyruvate Carboxylase [*Nicotiana tabacum]*
2	AT2G32220	Ribosomal L27e Protein Family	LOC107796761	60S Ribosomal Protein L27-like [Nicotiana tabacum]
3	VLN1	Villin-like 1	LOC107813523	Villin-2-like [*Nicotiana tabacum*]
4	PRF4	Profilin 4	LOC104211713	Profilin-like [Nicotiana sylvestris]
5	MLS	Malate Synthase	LOC107761798	Malate Synthase, Glyoxysomal-like [*Nicotiana tabacum*]
6	AGT	Alanine: Glyoxylate Aminotransferase	LOC107828015	Alanine—glyoxylate Aminotransferase 2 Homolog 3, Mitochondrial-like [*Nicotiana tabacum*]
7	ICL	Isocitrate Lyase	LOC107818961	İsocitrate Lyase [*Nicotiana tabacum*]
8	TPI	Triosephosphate Isomerase	LOC107804179	Triosephosphate Isomerase, Cytosolic-like [*Nicotiana tabacum*]
9	CP12-2	CP12 Domain-containing Protein 2	LOC107805556	Calvin Cycle Protein CP12-2, Chloroplastic-like [*Nicotiana tabacum*]
10	IPGAM1	Phosphoglycerate Mutase, 2,3-bisphosphoglycerate-independent	LOC107769473	2,3-bisphosphoglycerate-independent Phosphoglycerate Mutase [*Nicotiana tabacum*]
**B. sat-RNA (-) transcripts without HV**				
1	RPS5B	Ribosomal Protein 5B	LOC109223444	40S Ribosomal Protein S5 [*Nicotiana attenuata*]
2	EMB3010	Ribosomal Protein S6e	LOC104114214	40S Ribosomal Protein S6 [*Nicotiana tomentosiformis*]
3	ICL	Isocitrate Lyase	LOC107818961	İsocitrate Lyase [*Nicotiana tabacum*]
4	AT5G15200	Ribosomal Protein S4	RPS4	Ribosomal Protein S4 [*Nicotiana tabacum*]
**C. sat-RNA (+) transcripts with HV**				
1	FUT11	Fucosyltransferase 11	LOC107767349	Glycoprotein 3-alpha-l-fucosyltransferase A-like [*Nicotiana tabacum*]
2	CALS1	Callose Synthase 1	LOC109220900	Callose Synthase 2-like [*Nicotiana attenuata*]
3	ICL	Isocitrate Lyase	LOC107818961	İsocitrate Lyase [*Nicotiana tabacum*]
4	AT5G35530	Ribosomal Protein S3 Family Protein	RPS3	Ribosomal Protein S3 [*Nicotiana tabacum*]
**D. sat-RNA (-) transcripts with HV**				
1	PCK1	Phosphoenolpyruvate Carboxykinase 1	LOC107776000	Phosphoenolpyruvate Carboxykinase [Atp]-like [*Nicotiana tabacum*]
2	AT1G07070	Ribosomal Protein L35ae Family Protein	LOC107796252	60S Ribosomal Protein L35a-3-like [*Nicotiana tabacum*]
3	AT1G80750	Ribosomal Protein L30/L7 Family Protein	LOC104114515	60S Ribosomal Protein L7-1 [*Nicotiana tomentosiformis*]
4	ICL	Isocitrate Lyase	LOC107818961	İsocitrate Lyase [*Nicotiana tabacum*]
5	MLS	Malate Synthase	LOC107761798	Malate Synthase, Glyoxysomal-like [*Nicotiana tabacum*]

## Conclusions

This study provides a simple approach for isolating host proteins interacting with non-coding RNAs using a small, non-coding sat-RNA associated with CMV as a model. The method as described is amenable for recovering host proteins interacting with both (+) and (-)-strand polarity RNAs. We believe this approach can be applied to a wide range of RNAs associated with eukaryotic and prokaryotic pathogens. We observed a shift in the host proteome when (+) or (-)-sat-RNA transcripts were allowed to interact with total protein samples extracted from either healthy plants or plants challenged with HV. It is known that HV replicase that catalyzes the (-)-strand synthesis is distinct from that of (+)-strand [25]. Therefore, whether host proteins exclusively interacting with (+) or (-)-sat-RNA transcripts would contribute to this discriminatory role of HV replicase remains to be tested. For example, host factors putative citrate synthase glyoysomal precursor and eukaryotic initiation factor 4A-2 are present in total protein samples of HV-infected plants and found to interact exclusively (-)-sat-RNA but not with (+)-sat-RNA (Fig 3B). Therefore, inoculation of *N*. *benthamiana* or *A*. *thaliana* lines defective in the expression of either citrate synthase glycosomal precursor or eukaryotic initiation factor 4A-2 with CMV and its sat RNA followed by the analysis of (+) and (-)-sat-RNA progeny will help explore the possible roles played by these host proteins in HV-dependent replication of sat-RNA.
